# Unmanned aerial image dataset: Ready for 3D reconstruction

**DOI:** 10.1016/j.dib.2019.103962

**Published:** 2019-05-24

**Authors:** Mozhdeh Shahbazi, Patrick Ménard, Gunho Sohn, Jérome Théau

**Affiliations:** a Department of Geomatics Engineering, University of Calgary, Calgary, Alberta, Canada; b Centre de géomatique du Québec, Saguenay, Québec, Canada; c Department of Earth and Space Science and Engineering, York University, Toronto, Ontario, Canada; d Department of Applied Geomatics, Université de Sherbrooke, Sherbrooke, Québec, Canada

**Keywords:** Unmanned aerial vehicle, Structure from motion, Dense matching, Bundle adjustment, Stereo

## Abstract

Unmanned aerial vehicles (UAVs) have become popular platforms for collecting various types of geospatial data for various mapping, monitoring and modelling applications. With the advancement of imaging and computing technologies, a vast variety of photogrammetric, computer-vision and, nowadays, end-to-end learning workflows are introduced to produce three-dimensional (3D) information in form of digital surface and terrain models, textured meshes, rectified mosaics, CAD models, etc. These 3D products might be used in applications where accuracy and precision play a vital role, e.g. structural health monitoring. Therefore, extensive tests against data with relevant characteristics and reliable ground-truth are required to assess and ensure the performance of 3D modelling workflows. This article describes the images collected by a customized unmanned aerial vehicle (UAV) system from an open-pit gravel mine accompanied with additional data that will allow implementing and evaluating any structure-from-motion or photogrammetric approach for sparse or dense 3D reconstruction. This dataset includes total of 158 high-quality images captured with more than 80% endlap and spatial resolution higher than 1.5 cm, the 3D coordinates of 109 ground control points and checkpoints, 2D coordinates of more than 40K corresponding points among the images, a subset of 25 multi-view stereo images selected from an area of approximately 30 m × 40 m within the scene accompanied with a dense point cloud measured by a terrestrial laser scanner.

**Table:** Specifications table

Subject area	*Geomatics Engineering, Computer Science*
More specific subject area	*Photogrammetry, Computer vision, Pattern recognition*
Type of data	*TIFF images and text files*
How data was acquired	*Camera- Allied Vision Prosilica GE4900C* *Integrated GNSS system- Trimble R8* *Robotic total station- Trimble VX Spatial Station* *3D laser scanner- Faro Focus 3D*
Data format	*Raw, filtered and analyzed*
Experimental factors	*The data is filtered and analyzed to ensure quality for both sparse and dense 3D reconstruction from images*
Experimental features	*Robust feature matching, Sparse self-calibrating bundle adjustment, Point cloud registration*
Data source location	*Sherbrooke, Quebec, Canada*
Data accessibility	*Data is provided along with this article*
Related research article	[1]

**Table:** 

**Value of the data** Researchers in the fields of photogrammetry engineering, computer vision, and geography can use this dataset for: • Evaluating robust sparse matching algorithms • Evaluating Bundle Adjustment approaches • Evaluating multi-view stereo dense reconstruction algorithms • Evaluating incremental structure-from-motion techniques

## 1 Data

**Fig. 1:**
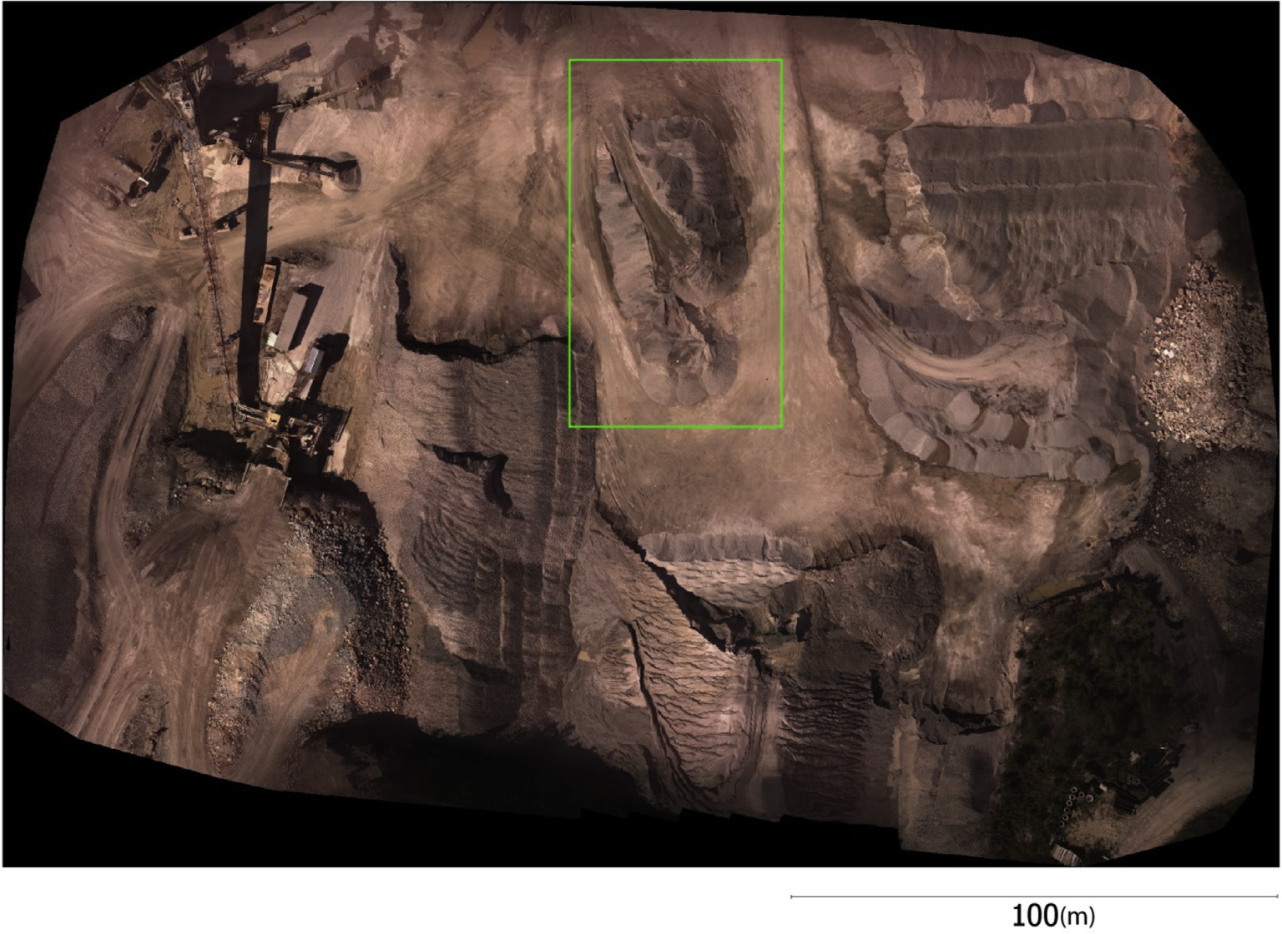
Top view of the imaged zone. The green rectangle identified the sub-zone from which a dense terrestrial laser scan is collected.

**Fig. 2:**
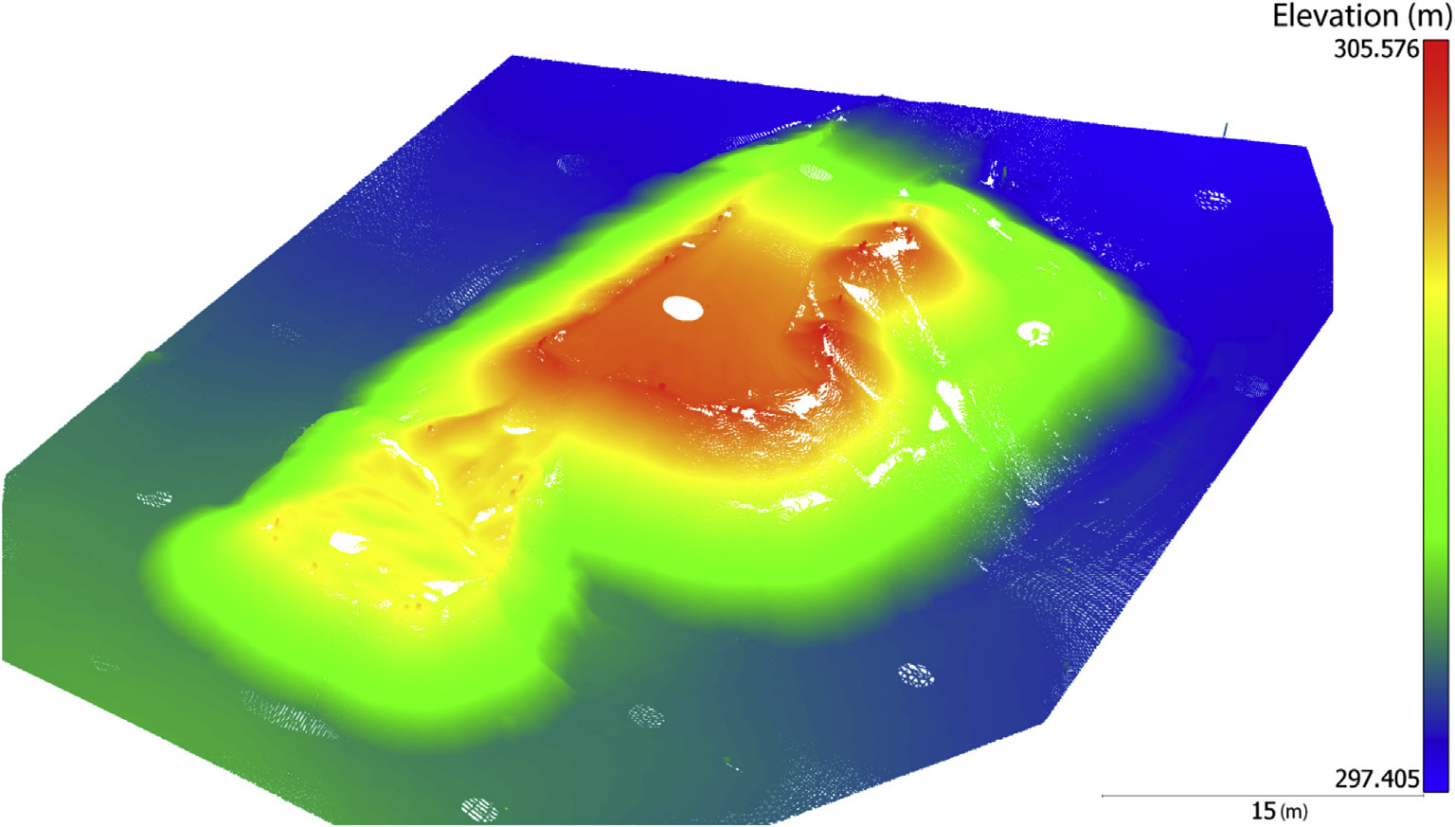
The dense point cloud collected by a terrestrial laser scanner.

This paper describes the images collected by a customized unmanned aerial vehicle (UAV) system from an open-pit gravel mine at approximate altitude of 80 m over an area of 150 m × 200 m (Fig. 1). These images are filtered and analyzed to produce additional data such as corresponding feature points (Table 1), which will allow implementing and evaluating any structure-from-motion or photogrammetric approach meant to perform sparse three-dimensional (3D) reconstruction. Accurate 3D control points and check points (Table 2) are provided to allow testing indirect geo-referencing techniques and validating the accuracy of 3D modelling. A dense point cloud captured by terrestrial lidar (Fig. 2) is provided to allow evaluating dense stereo or multi-view reconstruction techniques.

**Table 1:** Sample of observations of feature points extracted from raw images.

Image index	Point index	x-coordinate (pixel)	y-coordinate (pixel)
105	1898	1850.07	1648.52
85	1899	1714.68	919.37
58	1899	3933.77	25.07
69	3358	3046.02	1027.69
32	3358	49.29	2437.30
77	3358	3154.98	2323.47
21	3365	1734.01	997.65
20	3365	1545.64	51.57
21	3366	3023.48	513.69
22	3366	3225.28	1378.25
35	3366	3458.06	2655.06
115	3387	164.56	1100.38
74	3387	2215.44	1587.85
32	3387	284.94	3186.19
114	3387	8.63	461.73

**Table 2:** Sample of observations of ground control points measured by land surveying.

Point index	X-coordinate (m)	Y-coordinate (m)	Z-coordinate (m)
−5	193126.758	5025163.661	304.067
−6	193126.023	5025196.090	299.021
−7	193090.067	5025183.208	299.444
−8	193073.094	5025141.189	301.917
−9	193105.232	5025220.136	298.978
−10	193094.839	5025208.802	302.814

## 2 Experimental design, materials, and methods

### 2.1 Acquiring data

The imagery was acquired from an electric-powered helicopter (Responder, ING Robotic Aviation Inc., Ottawa, ON, Canada). A 16-Megapixels camera (GE4900C, Prosilica, Exton, PA, USA) was used to capture the images. It had an approximate sensor size of 36 × 24 mm, and a 35 mm F-adjustable lens. The camera was synchronized with the navigation sensors, a GPS-aided inertial navigation system (INS) (MIDGII, Microbotics Inc, Hampton, VA, USA). Control software was developed to store the data, to set the acquisition parameters of the camera and the INS (e.g., the exposure time, F-number, and triggering interval) and to geo-tag the images. This software solution was run on an ultra-small, single-board system (CoreModule 920, ADLINK, San Jose, CA, USA) on board the UAV and was remotely controlled from the ground station.

The data was acquired over several stockpiles in a gravel pit at Sherbrooke, QC, Canada. A simple software solution was developed and used to plan both the flight mission and the field survey. Several factors such as the geometric configuration of the photogrammetric network and the distribution of control data were taken into account to plan the data collection. A large number of targets were installed and measured using a high-precision, dual-frequency real-time kinematic (RTK) system (R8 GNSS System, Trimble, Sunnyvale, CA, USA) as well as a robotic total station (VX, Trimble, Sunnyvale, CA, USA). They can serve as ground control points (GCP).

Over one zone, ten dense 3D point clouds were collected by a terrestrial laser scanner (Focus 3D, FARO Technologies). To accurately co-register the individual scans, approximately 40 signalized targets with checkerboard patterns and reference spheres were installed and accurately measured. The readers are referred to [2] for further details about the data-acquisition experiments.

### 2.2 Data formatting

This dataset includes i) Total of 158 high-quality images captured via an industrial-grade camera equipped with a sensor of size 36 mm × 24 mm at pixel size of 7.4 μm and a 35 mm F-adjustable lens. The spatial resolution of this imagery is approximately 1.5 cm on the flat zones of the scene; ii) Approximate exterior orientation parameters of the images measured by the navigation sensors and filtered for gross errors; iii) The 3D coordinates of 109 ground control points collected by a high-precision, dual-frequency RTK GNSS system and a robotic total station as well as their 2D coordinates observed in the images; iv) 2D coordinates of more than 40K corresponding points among the images ensuring feasibility of accurate multi-view stereo analysis; v) A dense point cloud acquired by a terrestrial laser scanner over an area of approximately 30 m × 40 m within the scene that can be used as reference data for developing and testing high-density stereo matching techniques. The following paragraphs provide the formatting details of each component of this dataset.

#### 2.2.1 Images, direct geo-referencing data, time data

Uncompressed images are in Tiff format and are located in folder “RawImages”. Labels of the images start from 1 and incrementally continues to 158. Due to large volume, the images are available through an online storage space.^1^ The approximate exterior orientation parameters (EOPs) of images were recorded via a low-cost GNSS/IMU system. These observations were refined and are provided in the text file “DirectEOPs.txt”. This file consists of seven columns. The first column represents the image label. The second, third and fourth columns represent the 3D coordinates of the perspective center of the camera (3-vector C in Equation (1)). The coordinates are expressed in meters in the projected coordinate reference system, NAD 83/MTM Zone 7. The elevations are ellipsoidal heights. The fifth, sixth and seventh columns represent the rotation angles that can align the object coordinate system with the principal image coordinate system (Tait-Bryan Euler angles *ω, φ, κ* in Equation (2)). The unit of these angles is decimal degrees. Since images are captured in a high-overlapping sequence, one can use them in deterministic simultaneous localization and mapping algorithms or sequential structure-from-motion as well. To this end, one may need the timestamp of each image that corresponds to the exposure time of the image. The timestamps are provided in file “TimeStamps.txt” and are expressed in seconds up to a micro-second precision.

(1) $$\tilde{\text{u}}=K\left[\begin{array}{cc} R & -R\text{C} \end{array}\right]\tilde{\text{X}}$$

where:

$\tilde{\text{u}}$ : Homogeneous coordinates of the image point.

$\tilde{\text{X}}$: Euclideanly normalized homogeneous coordinates of the object point.

K: camera intrinsic calibration matrix.

C : coordinates of the perspective center of the camera in the world coordinate system.

R: rotation matrix describing the rotation of the image coordinate system wrt the world coordinate system defined as in Equation 2

(2) $$R=\left[\begin{array}{ccc} \cos\;\kappa & \sin\;\kappa & 0\\ -\sin\;\kappa & \cos\;\kappa & 0\\ 0 & 0 & 1 \end{array}\right]\left[\begin{array}{ccc} \cos\;\varphi & 0 & -\sin\;\varphi \\ 0 & 1 & 0\\ \sin\;\varphi & 0 & \cos\;\varphi \end{array}\right]\left[\begin{array}{ccc} 1 & 0 & 0\\ 0 & \cos\;\omega & \sin\;\omega \\ 0 & -\sin\;\omega & \cos\;\omega \end{array}\right]$$

where:

ω,φ,κ: rotation angles about the x-, y-, and z-axes of the world coordinate system.

#### 2.2.2 Corresponding points

Coordinates of corresponding points are provided in the text file “TiePoints.txt”. This text file includes four columns. The first column is the index of the image in which the point is observed (an integer between 1 and 158, inclusively). The second column is the index of the point. The third and the fourth columns are the 2D coordinates of the point in the image expressed in a coordinate system with its x-direction aligned with the image width, the y-direction aligned with the image height, and the origin at the top left corner of the image. The approximate precision of these image observations is 0.3 pixels, in both x- and y-directions. Equation (3) relates these coordinates (u) to the coordinates in the image principal coordinate system, a.k.a camera coordinate system (x), where the x-direction is aligned with the image width, the y-direction is aligned with the image height pointing towards the image top, and the origin is at the center of the image.

(3) $$\tilde{\text{u}}=K\tilde{\text{x}}\;\text{,}\;K^{-1}=\left[\begin{array}{ccc} 1 & 0 & \alpha_{x}\\ 0 & 1 & \alpha_{y}\\ 0 & 0 & -f \end{array}\right]\left[\begin{array}{ccc} s & 0 & -sw/2-x_{p}\\ 0 & -s & sh/2-y_{p}\\ 0 & 0 & 1 \end{array}\right]$$

where:

$\tilde{\text{x}}$: homogeneous coordinates of the point expressed in the image principal coordinate system.

s: pixel size in mm.

f: principal distance in mm.

w,h: image width and height in pixels.

$x_{p},y_{p}$: offsets of the principal point in mm.

$\alpha_{x},\alpha_{y}$: lens and sensor distortions as defined in Equation 4

(4) $$\begin{array}{l} \alpha_{x}=\left(x-x_{p}\right)\left(k_{1}r^{2}+k_{2}r^{4}+k_{3}r^{6}\right)+p_{1}\left(r^{2}+2\left(x-x_{p}\right)\right)+\\ \;\;\;\;\;\;\;\;+2p_{2}\left(x-x_{p}\right)\left(y-y_{p}\right)+b_{1}\left(x-x_{p}\right)+b_{2}\left(y-y_{p}\right)\\ \alpha_{y}=\left(y-y_{p}\right)\left(k_{1}r^{2}+k_{2}r^{4}+k_{3}r^{6}\right)+p_{2}\left(r^{2}+2\left(y-y_{p}\right)\right)+\\ \;\;\;\;\;\;\;\;+2p_{1}\left(x-x_{p}\right)\left(y-y_{p}\right) \end{array}$$

where:

$r^{2}={\left(x-x_{p}\right)}^{2}+{\left(y-y_{p}\right)}^{2}$: squared radial distance from the image center.

$k_{1},k_{2},k_{3}$: radial lens distortion coefficients.

$p_{1},p_{2}$: decentering lens distortion coefficients.

$b_{1},b_{2}$: affine sensor distortion coefficients.

#### 2.2.3 Accurately estimated 3D coordinates of the tie points, the EOPs, and the intrinsic calibration parameters

The 3D coordinates of the tie points and the EOPs of the images are estimated and refined through a robust, sparse, self-calibrating bundle adjustment approach. The 3D coordinates of the tie points are provided in the file “ObjectPoints.txt”, which includes four columns. The first column is the point index, and the last three columns are the coordinates of the point that are expressed in meters in the projected coordinate reference system, NAD 83/MTM Zone 7. The elevations are ellipsoidal heights. The estimated EOPs are included in the file “RefinedEOPs.txt”, which has the same format as the file “DirectEOPs.txt”. The intrinsic calibration parameters are included in the file “RefinedIOPs.txt”. The parameters are described as listed in the text file and they correspond to Equations (3), (4)).

#### 2.2.4 Ground control points

The ground-truth coordinates of 109 tie points are measured via surveying tools and provided in file “GCPs.txt”. This file has the same format as file “ObjectPoints.txt”. Considering the surveying method, an approximate precision of 2–5 mm is acceptable for these observations. The GCPs are labeled with negative integer numbers to allow them to be easily identified in tie-point observation file “TiePoints.txt”.

#### 2.2.5 Image pairs and ground-truth dense point cloud

If one is interested in testing a dense multi-view stereo reconstruction approach on the images of this dataset, our recommendation is using a subset of images whose indices are provided in the text file “DenseMatching.txt”. These images cover a stockpile, which is accurately and densely surveyed with a 3D terrestrial laser scanner (Fig. 2). Therefore, after dense matching, one can evaluate the generated depth maps against this point cloud. The point-cloud data is provided in the file “LaserScan.xyz”, which includes three columns corresponding to the 3D coordinates of the scanned points in NAD 83/MTM Zone 7. The elevations are ellipsoidal heights.

### 2.3 Pre-processing

View-clustering is performed, and image pairs with sufficient overlap and proper geometric configuration are identified. Initial image matching is performed using a modified SIFT algorithm. A robust method based on an evolutionary algorithm is used to estimate the two-view epipolar geometry of image pairs and identify the inlier corresponding points [3]. Then, a sequential approach is applied to convert two-view parameters of epipolar-geometry between image pairs first to relative orientation parameters (ROPs) and, then, to exterior orientation parameters. The initial 3D coordinates of the tie-points are reconstructed as well. Initial estimates are optimized using robust sparse self-calibrating bundle adjustment, as a result of which remaining large errors among the observations of the corresponding points are removed as well [4]. After this process, the average frequency of the tie points (i.e., the number of images a tie-point is observed in) is 11 images, and the average density of the images (i.e., the number of tie points observed in an image) is 261 points. The total number of observations is 41346 points. For a subset of 25 images taken over an area of 30 m × 40 m (one stockpile), the estimated intrinsic calibration parameters of the camera and the EOPs of the images can be used to rectify corresponding image pairs to normal stereo images [1]. The ground-truth point cloud provided in this dataset is generated by co-registering ten scans. Registration is performed using a total of 43 accurately surveyed signalized targets (spheres and cross-check patterns). The point cloud is comprised of over 8,300,000 points.

## Footnotes

1 https://drive.google.com/open?id=1nFag4gJBDLiDD6muD-YkbRZocawbUBBR.
