# Supplementary material for: Unmanned aerial image dataset: Ready for 3D reconstruction
Source: Data Brief. 2019 May 24;25:103962. doi: 10.1016/j.dib.2019.103962 (PMC6554229; doi:10.1016/j.dib.2019.103962)
Supplement: Multimedia component 1 [file mmc1.pdf]

Conflict of Interest and Authorship Conformation Form

Please check the following as appropriate:

■ All authors have participated in (a) conception and design, or analysis and interpretation of the data; (b) drafting the article or revising it critically for important intellectual content; and (c) approval of the final version.

■ This manuscript has not been submitted to, nor is under review at, another journal or other publishing venue.

■ The authors have no affiliation with any organization with a direct or indirect financial interest in the subject matter discussed in the manuscript
